# Self-expandable metallic stenting as a bridge to elective surgery versus emergency surgery for acute malignant right-sided colorectal obstruction

**DOI:** 10.1186/s12893-020-00993-4

**Published:** 2020-12-10

**Authors:** Bing Li, Shi-Lun Cai, Zhen-Tao Lv, Ping-Hong Zhou, Li-Qing Yao, Qiang Shi, Zhi-Peng Qi, Di Sun, Ayimukedisi Yalikong, En-Pan Xu, Jian-Min Xu, Yun-Shi Zhong

**Affiliations:** 1 Endoscopy Center, Zhongshan Hospital of Fudan University, 180 Fenglin Road, Shanghai, 200032 China; 2 Endoscopy Research Institute of Fudan University, Shanghai, 200032 China; 3 Department of General Surgery, Zhongshan Hospital, Fudan University, Shanghai, 200032 China

**Keywords:** Right-sided colon cancer, Obstruction, Self-expandable metallic stent, Liver metastases

## Abstract

**Background:**

The use of a self-expandable metallic stent (SEMS) as a bridge to surgery has increased for patients with obstructing colorectal cancer. However, relatively few reports have compared SEMS as a bridge to elective surgery for acute malignant obstruction of the right-sided colon (MORC) vs. emergency surgery (ES). This study aimed to evaluate the benefits of elective surgery after SEMS placement vs. ES for patients (including stage IV cases) with acute MORC.

**Methods:**

Patients with acute MORC who underwent radical resection for a primary tumour from July 2008 to November 2016 at Zhongshan Hospital of Fudan University were retrospectively enrolled. Postoperative short-term outcomes, progression-free survival (PFS), and overall survival (OS) were compared between the SEMS and ES groups.

**Results:**

In total, 107 patients with acute MORC (35 in the SEMS group and 72 in the ES group) were included for analysis. The Intensive Care Unit admission rate was lower (11.4% vs. 34.7%, *P* = 0.011), the incidence of complications was reduced (11.4% vs. 29.2%, *P* = 0.042), and the postoperative length of hospitalisation was significantly shorter (8.23 ± 6.50 vs. 11.18 ± 6.71 days, *P* = 0.033) for the SEMS group. Survival curves showed no significant difference in PFS (*P* = 0.506) or OS (*P* = 0.989) between groups. Also, there was no significant difference in PFS and OS rates between patients with stage II and III colon cancer. After colectomy for synchronous liver metastases among stage IV patients, the hepatectomy rates for the SEMS and ES groups were 85.7% and 14.3%, respectively (*P* = 0.029). The hazard ratio for colectomy alone vs. combined resection was 3.258 (95% CI 0.858–12.370; *P* = 0.041).

**Conclusion:**

Stent placement offers significant advantages in terms of short-term outcomes and comparable prognoses for acute MORC patients. For synchronous liver metastases, SEMS placement better prepares the patient for resection of the primary tumour and liver metastasis, which contribute to improved survival.

## Background

Colorectal cancer (CRC) is the fourth most commonly diagnosed cancer and the second leading cause of cancer-related death in both males and females [1]. Approximately 8–13% of patients with advanced colon cancer present with an obstruction of the large bowel [2–4]. A self-expandable metallic stent (SEMS) is widely used for obstructive left-sided colon cancer to allow for an easy endoscopic approach to the lesion and to facilitate patient recovery from the acute status with reduced risks of postoperative complications and mortality [5–7]. However, fewer than 10% of reported cases of colonic stenting have involved the right colon [8].

Some studies have reported insertion of a SEMS for acute malignant obstruction of the right-sided colon (MORC) could benefit patients with severe comorbidities, advanced age, or complete obstruction [9, 10]. Moreover, the technical success rate in experienced centres has improved to > 96%, similar to that reported for stenting of distal colon lesions [11]. Thus, the present retrospective study included more cases than previous reports of the advantages of SEMS as a bridge to elective surgery as compared to emergency surgery (ES) for CRC patients with a proximal malignant obstruction of the large bowel. In addition, bowel obstruction is often accompanied by distant metastasis, as the liver is the most common site of CRC metastasis [4, 12]. Here, we report our experience and results with the use of colonic stenting of patients, including those with stage IV CRC, an area of the published data that remains severely limited. Therefore, the aim of the present study was to evaluate the benefits of elective surgery after SEMS placement vs. ES for patients (including stage IV cases) with acute MORC.

## Methods

### Ethics statement

The study protocol was approved by the Institutional Review Board of Zhongshan Hospital and conducted in accordance with the tenets of the Declaration of Helsinki. Informed consent was obtained from all patients prior to treatment. This retrospective observational study was conducted in accordance with the Strengthening the Reporting of Observational studies in Epidemiology (STROBE) guidelines [13].

### Patients

The study cohort was limited to patients with acute right-sided bowel obstruction caused by malignant CRC who underwent radical resection for the primary tumour from July 2008 to November 2016 at Zhongshan Hospital of Fudan University (Shanghai, China). Right-sided colon cancer was defined as any tumour arising in the cecum, ascending colon, hepatic flexure or transverse colon. MORC was clinically defined as symptoms of abdominal pain, distension, vomiting, and no passage of stool or flatus, and radiologically defined as severe dilatation of the proximal colon due to suspected colon cancer by abdominal X-ray and/or contrast-enhanced computed tomography (CT). Radical surgery was performed if no distant metastasis was observed either pre- or intra-operatively. However, if distant metastasis was found, radical resection was performed for the primary tumour, while sites of metastasis were treated by synchronous or two-stage resection, or other non-surgical treatments.

The patients were assigned to one of two groups: the ES group, which consisted of patients who underwent radical resection within 24 h after visiting the hospital and received no other treatments for primary causes, or the SEMS group, which consisted of patients who underwent colonic stent placement followed by surgery within 2 weeks after stent placement. The strategy to choose ES or SEMS placement as a bridge to surgery was mainly based on the following considerations: (1) the tumor locations differed significantly between the SEMS and ES groups, as stenting was not appropriate for an obstruction in the cecum; (2) low-pressure enema intestinal cleaning could be completed to facilitate stricture visualization and stent placement; (3) there were no signs of peritonitis or perforation in the SEMS group; (4) if ES was considered too risky or when the disease was very advanced and palliation was needed, stent placement as a bridge to surgery was considered; and (5) the final choice of treatment (ES or SEMS placement) was mostly dependent on a consensus among the surgeons, the endoscopists and the patient or patient’s family.

### Procedure

All stent placement procedures were performed by experienced endoscopists at the Endoscopy Centre of Zhongshan Hospital with experience and competence in both colonoscopy and fluoroscopic techniques and who performs colonic stenting on a regular basis. Briefly, the stent placement procedures consisted of four steps: (1) determining the site and aetiology of the acute bowel obstruction by colonoscopy combined with fluoroscopy; (2) a hydrophilic biliary guidewire was introduced through the tumour beyond the point of obstruction; (3) injection of water-soluble contrast medium proximally to the stricture; and (4) insertion and placement of suitable stents under fluoroscopic guidance. The immediate escape of air and liquid faeces through the stent indicated successful decompression.

Afterward, a series of examinations, including chest X-ray, abdominal ultrasound or abdominal CT, and blood tests, were performed. At 7 to 14 days after the colon obstruction was relieved, mechanical bowel preparation was performed using polyethylene glycol or sodium phosphate and one-stage surgery.

### Staging assessment and follow-up

Pathological tumour-node-metastasis staging was performed in accordance with the guidelines of the Union for International Cancer Control, eighth edition. For all patients, routine clinical follow-up data were obtained. CT, abdominal ultrasound, chest X-ray, and blood tests were performed every 3 months for the 1st year and then every 6 months thereafter. Colonoscopic surveillance was performed every 6 months for the 1st year and then once per year thereafter. Diagnoses of relapse and metastasis were based on imaging studies and biopsy, if necessary. The follow-up period was defined as the date of surgery to either the date of death or August 2018, whichever occurred first.

### Data collection and analysis

In addition to clinicopathological data (i.e., age, sex, tumour characteristics, histopathology and surgical information), short-term postoperative outcomes and long-term prognoses were collected for analysis. The short-term postoperative outcomes mainly consisted of admission to the Intensive Care Unit (ICU), adverse events, and mortality within 30 days after surgery. The primary endpoints of long-term outcomes were progression-free survival (PFS) and overall survival (OS). Data were primarily obtained from medical records. For patients who had moved away, attempts were made to obtain outcome details by telephone contact with the patient or a family member.

Comparisons between groups were performed using the Student's *t*-test, chi-squared test, or Fisher’s exact test and rank-sum test, as appropriate. Kaplan–Meier curves were constructed to analyse rates of survival, recurrence, and metastasis. The log-rank test was used to evaluate the significance of differences between curves. All statistical analyses were performed using SPSS for Windows, version 16.0. (SPSS Inc., Chicago, IL, USA). A probability (*P*) value of < 0.05 was considered statistically significant.

## Results

### Baseline characteristics

From July 2008 to November 2016, 107 patients with acute MORC (35 patients in the SEMS group and 72 in the ES group) underwent radical resection at Zhongshan Hospital. The median patient age was 66 (range 23–94) years. As shown in Table 1, there were no major differences in baseline and oncologic characteristics, with the exception of tumour location, between the SEMS and ES groups. No stent migration or perforation was observed, although one patient experienced re-obstruction after initial successful stenting. As of the last follow-up on August 2018, the overall median follow-up duration was 35 (range 0.1–120) months. Of the 107 patients, 13 (12.1%) were lost to follow-up. However, there was no significant difference in the rate of patients lost to follow-up between the SEMS and ES groups (11.4% [4/35] vs 12.5% [9/72], respectively; *P* > 0.99).

**Table 1:** Baseline and oncologic characteristics of the included patients

	SEMS group (n = 35)	Emergency group (n = 72)	P
Baseline characteristics			
Age, y			0.144
Median	66	67	
Range	24–92	23–94	
Sex, no. (%)			0.131
Male	21 (60.0%)	32 (44.4%)	
Female	14 (40.0%)	40 (55.6%)	
Comorbidity, no. (%)			
Hypertension	10 (28.6%)	14 (19.4%)	0.288
Diabetes mellitus	5 (14.3%)	11 (15.3%)	0.893
Cardiovascular disease	3 (8.6%)	6 (8.3%)	1.000
Pulmonary disease	2 (5.7%)	2 (2.8%)	0.596
Neurologic disease	1 (2.9%)	2 (2.8%)	1.000
Other malignancy	2 (5.7%)	3 (4.2%)	0.661
Renal disease	0 (0%)	1 (1.4%)	1.000
Oncologic characteristics			
Tumor size, mean (± SD), cm	7.71 ± 3.70	5.85 ± 2.58	0.216
Tumor location, no. (%)			0.022
Cecum	0 (0%)	11 (15.3%)	
Ascending colon	12 (34.3%)	26 (36.1%)	
Hepatic flexure	7 (30.0%)	17 (23.6%)	
Transverse colon	16 (45.7%)	18 (25.0%)	
Pathology, no. (%)			0.893
Adenocarcinoma	30 (85.7%)	61 (84.7%)	
Well differentiated	2 (5.7%)	3 (4.2%)	
Moderately differentiated	26 (74.3%)	54 (75.0%)	
Poorly differentiated	2 (5.7%)	4 (5.5%)	
Mucinous	5 (14.3%)	11 (15.3%)	
Lymphovascular involvement, no. (%)			
Yes	13 (37.1%)	17 (23.6%)	0.144
No	22 (62.9%)	55 (76.4%)	
pTNM stage			0.240
II	16 (45.7%)	31 (43.1%)	
III	12 (34.3%)	34 (47.2%)	
IV	7 (20.0%)	7 (9.7%)	

*SEMS* self-expandable metal stents

### Characteristics of the procedures and postoperative short-term outcomes

The characteristics of the surgical procedures and short-term postoperative outcomes of the two groups are shown in Table 2. Although open surgery was the primary approach, laparoscopic procedures were performed more frequently in the SEMS group than the ES group (11.4% vs. 0%, respectively; P = 0.010). In regard to intraoperative findings, the incidence of ascites was greater in the ES group than the SEMS group (52.8% vs. 20.0%, respectively; P = 0.001), while perforation occurred in four (5.6%) patients in the ES group. The need for intraoperative transfusion tended to be lower in the SEMS group than the ES group, but the difference was not statistically significant (2.9% vs. 13.9%, respectively; P = 0.098). In the SEMS group, jejunostomy was performed for one patient, as partial duodenectomy was required due to intraoperative findings that the tumor had invaded the duodenum.

**Table 2:** Characteristics of the surgical procedures and postoperative short-term outcomes

	SEMS group (n = 35)	Emergency group (n = 72)	P
Operation method, no. (%)			0.010
Laparoscopy	4 (11.4%)	0 (0%)	
Open	31 (88.6%)	72 (100%)	
Operation findings, no. (%)			
Ascites	7 (20.0%)	38 (52.8%)	0.001
Perforation	0 (0%)	4 (5.6%)	0.301
Stoma formation	1 (2.9%)	0 (0%)	0.327
Transfusion, no. (%)	1 (2.9%)	10 (13.9%)	0.098
Blood loss, mean (± SD), ml	70.00 ± 39.92	77.22 ± 50.94	0.414
Operation time, mean (± SD), min	118.14 ± 29.95	147.14 ± 43.77	0.052
Positive margin, no. (%)	0 (0%)	0 (0%)	/
No. of retrieved LNs, mean (± SD)	21.09 ± 9.89	19.96 ± 9.53	0.766
No. of metastatic LNs, mean (± SD)	1.86 ± 3.91	1.89 ± 2.69	0.573
ICU stay, no. (%)	4 (11.4%)	25 (34.7%)	0.011
ICU stay time, mean (± SD), day	4.25 ± 2.87	3.96 ± 2.81	0.882
Postoperative complication, no. (%)	4 (11.4%)	21 (29.2%)	0.042
Wound infection	1 (2.9%)	5 (6.9%)	0.661
Pneumonic infection	2 (5.7%)	11 (15.3%)	0.217
Anastomotic leakage	0 (0%)	3 (4.2%)	0.549
Gastric retention	0 (0%)	1 (1.4%)	1.000
MODS	0 (0%)	1 (1.4%)	1.000
AHF	1 (2.9%)	0 (0%)	0.327
30-days mortality, no. (%)	0 (0%)	1 (1.8%)	1.000
Hospital stay, mean (± SD), day	8.23 ± 6.50	11.18 ± 6.71	0.033

*SEMS* self-expandable metal stents, *LN* lymph node, *ICU* intensive care unit, *MODS* multiple organ dysfunction syndrome, *AHF* acute heart failure

The postoperative ICU admission rate was significantly lower in the SEMS group than the ES group (11.4% [4/35 vs. 34.7% [25/72], respectively; *P* = 0.011). Moreover, the complication rate was significantly lower in the SEMS group than the ES group (11.4% [4/35] vs. 29.2% [21/72], respectively; *P* = 0.042). The most common postoperative complications in both groups were wound infection, pulmonary infection, and anastomotic leakage, but there was no significant difference in the incidence of complications between the two groups (*P* > 0.05). One patient in the ES group died due to multiple organ dysfunction syndrome on postoperative day 3. Moreover, the average duration of postoperative hospitalization was significantly shorter in the SEMS group than the ES group (8.23 ± 6.50 vs. 11.18 ± 6.71 days, respectively; *P* = 0.033). After excluding tumors located in the cecum from the ES group, the characteristics of the surgical procedures and postoperative short-term outcomes of the two groups were compared. The results in Additional file 1: Table S1 show the advantages of stent placement in terms of lower ICU admission rate, reduced complication rates, and shorter postoperative hospital stays.

### Long-term outcomes of all populations in the SEMS and ES groups

**Fig. 1:**
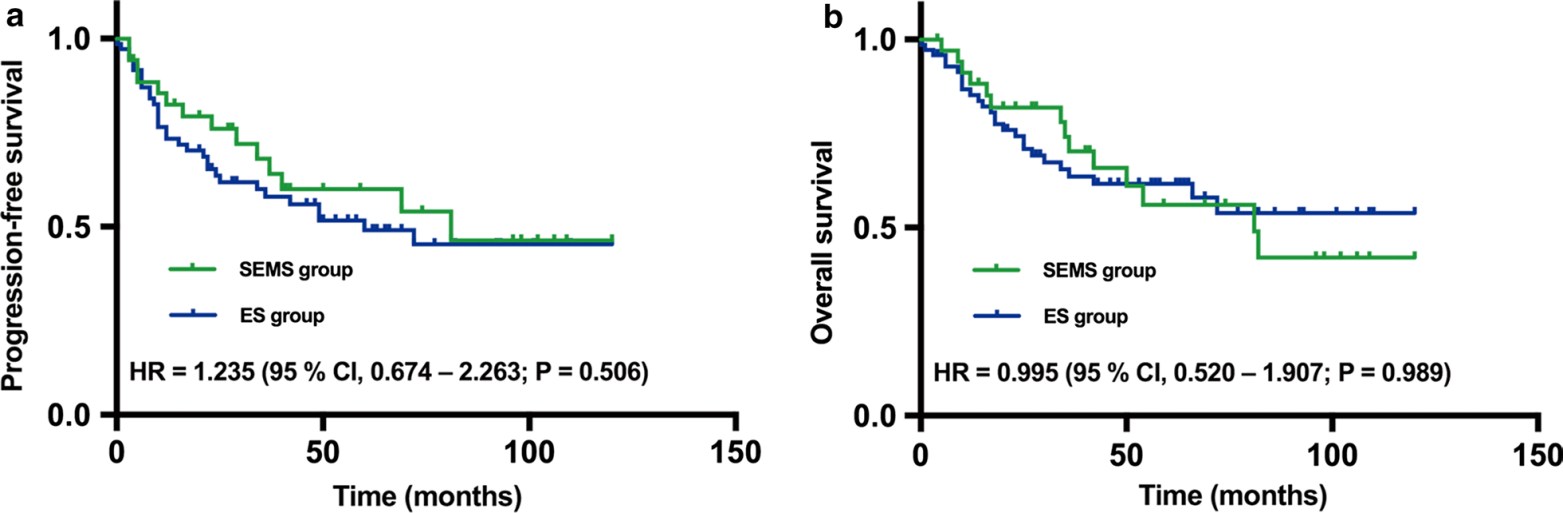
**a** PFS and **b** OS outcomes of the SEMS and ES groups

Kaplan–Meier curves of PFS for all patients are presented in Fig. 1a. The hazard ratio (HR) for PFS between the ES vs. SEMS groups was 1.235 (95% confidence interval [CI] 0.674–2.263; *P* = 0.506). The 5-year PFS rate was greater in the SEMS group than the ES group (54.0% [95% CI 34.20–73.80%] vs. 49.1% [95% CI 35.97–62.23%], respectively). The Kaplan–Meier curves of OS for all patients are presented in Fig. 1b. The HR for OS between the ES and SEMS group was 0.995 (95% CI 0.520–1.907; *P* = 0.989). The 5-year OS rate was lower in the SEMS group than the ES group (56.0% [95% CI 36.40–75.60%] vs. 61.6% [95% CI 49.25–73.95%], respectively).

### Subgroup analyses based on tumor stage

#### Comparison of stage II and III disease between the SEMS and ES groups

During the follow-up period, disease progression, defined as local site recurrence and distant metastasis, was observed in 27 (29.0%) patients with stage II and III disease. There was no significant difference in the distant metastasis rate between the SEMS and ES groups (21.4% [6/28] vs. 18.5% [12/65], respectively; *P* = 0.740) or in the rate of local relapse (7.1% [2/28] vs*.* 10.8% [7/65], respectively; *P* = 0.719). At the time of analysis, a total of 31 (33.3%) patients died during the follow-up period. However, there was no significant difference in the mortality rate between the SEMS and ES groups (32.1% [9/28] vs*.* 33.8% [22/65], respectively; *P* = 0.873) (Table 3).

**Table 3:** Long-term prognosis outcomes on patients with stage II and stage III disease in the SEMS group and emergency group

	SEMS group (n = 28)	Emergency group (n = 65)	P
Distant metastasis, no. (%)	6 (21.4%)	12 (18.5%)^a^	0.740
Liver	1 (3.6%)	7 (10.8%)	0.427
Others	5 (17.9%)	8 (12.3%)	0.522
Lungs	2 (7.1%)	4 (6.2%)	
Peritoneum	2 (7.1%)	3 (4.6%)	
Bone	0 (0%)	1 (1.5%)	
Adrenal gland	1 (3.6%)	0 (0%)	
Local site relapse, no. (%)	2 (7.1%)	7 (10.8%)	0.719
Death	9 (32.1%)	22 (33.8%)	0.873

^a^Two patient developed liver metastases and lungs metastases, and one patient developed liver metastases and bone metastases at the same time during follow-up

**Fig. 2:**
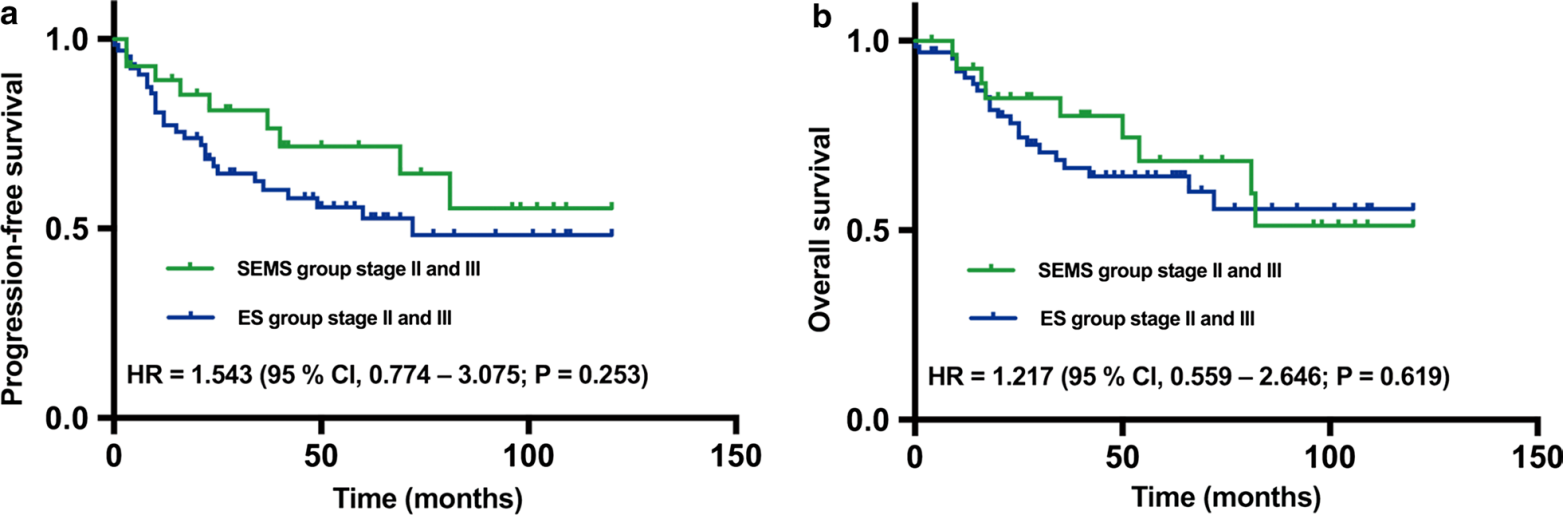
**a** PFS and **b** OS of patients with stage II and III disease in the SEMS and ES groups

PFS curves of the 93 patients with stage II and III disease are shown in Fig. 2a. As the HR for the ES vs*.* SEMS groups was 1.543 (95% CI 0.774–3.075; *P* = 0.253), the 5-year PFS rate was 64.5% (95% CI 43.53–85.47) for the SEMS group and 52.6% (95% CI 38.64–66.52) for the ES group. The Kaplan–Meier curves of OS are presented in Fig. 2b. The HR for the ES vs. SEMS group was 1.217 (95% CI 0.559–2.646; *P* = 0.619), when the 5-year OS rate was 68.2% (95% CI 47.82–88.58) for the SEMS group and 64.2% (95% CI 51.46–76.94) for the ES group.

#### Long-term outcomes of patients with stage IV disease

Table 4 shows the clinicopathological characteristics and long-term prognostic outcome data of 14 patients with stage IV colon cancer in the two groups. The SEMS and ES groups both had seven CRC patients with synchronous liver metastases. After colectomy, the synchronous or two-stage hepatectomy rates was significantly greater in the SEMS group than the ES group (85.7% [6/7] vs. 14.3% [1/7], respectively; *P* = 0.029). The remaining patients received chemotherapy, transcatheter arterial chemoembolisation or other palliative treatments.

**Table 4:** The clinicopathological characteristics and long-term prognosis outcomes data on patients with stage IV disease

No	SEMS	Patient		Lesion						Outcomes	
		Age, years	Sex	location	Pathology	Lymphovascular involvement	Metastatic site	Synchronous or two-stage hepatectomy	Date of colectomy	Recurrence/metastasis	Death
#1	Yes	63	F	Transverse colon	Adenocarcinoma	Yes	Liver	Yes	Aug 2009	Yes	Jan 2013
#2	Yes	54	M	Ascending colon	Adenocarcinoma	No	Liver	No, chemotherapy	Mar 2010	/	Aug 2010
#3	Yes	67	M	Ascending colon	Adenocarcinoma	Yes	Liver	Yes	Sep 2010	Loss to follow-up	/
#4	Yes	66	M	Transverse colon	Mucinous	No	Liver	Yes	Oct 2011	Yes	Jul 2014
#5	Yes	73	M	Ascending colon	Adenocarcinoma	Yes	Liver	Yes	Aug 2012	Yes	Aug 2013
#6	Yes	64	M	Hepatic flexure	Adenocarcinoma	No	Liver	Yes	May 2013	Yes	Jun 2016
#7	Yes	47	M	Ascending colon	Adenocarcinoma	No	Liver	Yes	Jan 2015	No	No
#8	No	49	M	Ascending colon	Adenocarcinoma	No	Liver	Yes	Dec 2010	No	No
#9	No	64	F	Transverse colon	Adenocarcinoma	Yes	Liver	No, chemotherapy	Dec 2010	/	Aug 2015
#10	No	64	F	Ascending colon	Adenocarcinoma	Yes	Liver	No, chemotherapy	Feb 2013	/	Nov 2013
#11	No	77	F	Ascending colon	Adenocarcinoma	No	Liver	No	Oct 2013	Loss to follow-up	/
#12	No	64	F	Cecum	Adenocarcinoma	Yes	Liver	No	Jan 2014	/	Jun 2014
#13	No	62	F	Cecum	Adenocarcinoma	No	Liver	No, TACE	Aug 2015	/	Nov 2015
#14	No	48	F	Cecum	Adenocarcinoma	Yes	Liver	No	May 2016	/	Dec 2016

*SEMS* self-expandable metal stents, *TACE* transcatheter arterial chemoembolization

**Fig. 3:**
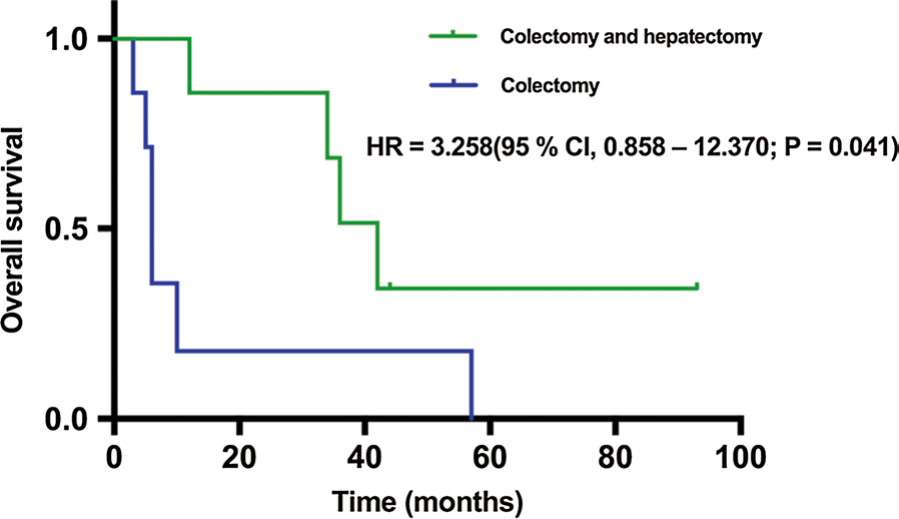
OS curves of patients with stage IV disease treated by colectomy combined with hepatectomy and colectomy alone

To determine the advantages of different therapeutic regimens, survival outcomes of patients who underwent colectomy only vs. colectomy combined with hepatectomy were compared. Kaplan–Meier curves of OS are shown in Fig. 3. The data showed that median OS was superior for those who underwent combined resection as compared to colectomy alone (42 vs. 6 months, respectively), and the HR for colectomy only *vs* combined resection was 3.258 (95% CI 0.858–12.370; P = 0.041).

## Discussion

In fact, since Campbell et al. reported the efficacy and safety of successful SEMS placement in MORC patients in 1997 [14], this technique has gained more and more attention. Repici et al. reported that the success rate for SEMS insertion for right-sided malignant colonic obstruction was 95% (20/21), with resolution of obstructive symptoms and no immediate complications in 85% of cases (17/20) [15]. Similarly, another recent study reported a success rate of 87.5% and symptom relief rate of 100% with no immediate complications [16]. In the present study, re-obstruction as a long-term complication occurred in only one patient. Collectively, these findings confirm the feasibility of SEMS placement for treatment of MORC.

Considering the higher morbidity and mortality rates as compared with elective surgery [17, 18], successful SEMS placement can provide sufficient preoperative preparation for patients with acute malignant colorectal obstruction prior to open or laparoscopic one-stage colectomy [19, 20]. In the present study, 35 patients initially underwent SEMS placement as a bridge to elective surgery, while 72 patients underwent ES. Although open colectomy accounted for the majority of surgeries, laparoscopic colectomy, as opposed to ES, tended to be implemented in the SEMS group. As compared to open surgery, the advantages of laparoscopic approach include faster recovery and lower postoperative morbidity in the SEMS group. However, prior to 2016, colorectal surgeons at our center had limited experience with the laparoscopic approach, which explains the low rate of laparoscopic surgery, as it takes time to incorporate a new technology. In regard to the intra-operative findings, the incidence of ascites was greater in the ES group (52.8%, 38/72) than the SEMS group (20.0%, 7/35), indicating that the physical status of patients in the SEMS group was better than that of the ES group. Likewise, in the present study, short-term outcomes were better in the SEMS group than the ES group. In addition, the ICU admission rate was significantly lower in the SEMS group (11.4%, 4/35) than the ES group (34.7%, 25/72), suggesting that the main advantages of stent placement were a reduced incidence of postoperative complications and shorter hospital stay. Other studies reported similar conclusions. One study reported shorter postoperative hospital stays and time to resume oral food intake in the SEMS group, suggesting better recovery from surgery [16].

The long-term prognosis of stent placement for MORC as a bridge to surgery was an important focus of the present study. Considering the differences in treatment methods and survival results, the PFS and OS rates were separately compared between the SEMS and ES groups in terms of stage IV disease and other stages. The results showed no significant differences in PFS and OS rates between patients with stage II and III colon cancer. A multicentre retrospective study also indicated that the long-term oncologic outcome of the SEMS group was similar or slightly better than that of the ES group among all patients with stage II or III colon cancer [21]. According to a meta-analysis of 11 studies, which included 1136 patients with left-sided or right-sided obstructive colon cancer, stenting as a bridge to surgery was oncologically comparable to ES with respect to OS, disease-free survival, and recurrence [22]. Moreover, Li et al. and Gianotti et al. reported improved survival of the SEMS group throughout the follow-up period [23, 24].

For patients with CRC, the liver is the most common site of metastasis and hepatic metastasis during the course of disease is the main cause of death [25, 26]. Of the 93 patients with stage II or III disease in the present study, liver metastasis occurred in eight (8.6%) after surgery. In cases of synchronous CRC liver metastases (CRCLM), the prognosis of untreated patients is poor, as fewer than 30% had survived at 1 year and fewer than 5% at 5 years after diagnosis [27]. Surgical resection is the most effective treatment for CRCLM, as the 5-year survival rate after liver resection reportedly ranges from 44 to 57% [28, 29]. In the present study, 14 patients had right-sided colorectal obstructions with synchronous metastasis. In the SEMS group, six (95.7%) of seven patients underwent resection of the primary tumour and the metastatic sites of the liver, while only one patient in the ES group underwent combined resection and the other seven underwent colectomy for severe obstruction. Thus, we inferred that SEMS improved the suitability of patients with stage IV disease for radical resection. Among the patients who underwent liver surgery, median survival was 42 months, which is comparable to the survival duration of 36 to 57 months in other reports [29, 30]. Based on these survival data of different treatment regimens, it is obvious that patients could benefit from resection of both the primary tumour and sites of metastasis.

There were several limitations to this study. First, in terms of baseline characteristics, SEMS was not employed in the cecum due to differences in tumour location. The main reason for this imbalance is that stent placement in the cecum of the right-sided colon is more technically challenging, as the stent should preferably extend beyond the stricture at both ends by 1.5–2 cm. Of course, the results of the present study were limited by the relatively small number of patients, especially those with liver metastases, and the single-centre retrospective study design. The small sample number was also a limit to the research on learning curve of right colon stenting. Nonetheless, future studies with larger numbers of subjects and longer follow-up periods are warranted.

## Conclusion

In conclusion, stent placement as a bridge to surgery followed by selective surgery provides significant advantages in terms of short-term outcomes as compared to ES, but with comparable prognoses for patients with acute MORC. For patients with synchronous liver metastases, stent placement provides more opportunities for resection of the primary tumour and sites of metastasis in the liver, which can further improve survival.

## Supplementary Information

**Additional file 1: Table S1.** Characteristics of the surgical procedures and postoperative short-term outcomes after excluding tumors in the cecum.

## Data Availability

The datasets used and analysed during this study are available from the corresponding author upon reasonable request.

## Abbreviations

SEMS: Self-expandable metal stents
MORC: Malignant obstruction of right-sided colon
PFS: Progression-free survival
OS: Overall survival
ES: Emergency surgery
ICU: Intensive Care Unit
HR: Hazard ratio
CRC: Colorectal cancer
CT: Computed tomography
MODS: Multiple organ dysfunction syndrome
AHF: Acute heart failure
CI: Confidence interval
TACE: Transcatheter arterial chemoembolization
ESGE: European Society of Gastrointestinal Endoscopy
CRCLM: CRC liver metastases
