# Supplementary material for: Self-expandable metallic stenting as a bridge to elective surgery versus emergency surgery for acute malignant right-sided colorectal obstruction
Source: BMC Surg. 2020 Dec 10;20:326. doi: 10.1186/s12893-020-00993-4 (PMC7727111; doi:10.1186/s12893-020-00993-4)
Supplement: Supplementary file 1 — Additional file 1: Table S1. Characteristics of the surgical procedures and postoperative short-term outcomes after excluding tumors in the cecum. [file 12893_2020_993_MOESM1_ESM.docx]

| **Additional Table S1.** **Characteristics of the** **surgical procedures and postoperative short-term outcomes after excluding tumors in the cecum** | | | |
| --- | --- | --- | --- |
|  | **SEMS group (n=35)** | **Emergency group (n=61)** | **P** |
| Operation method, no. (%) |  |  | 0.016 |
| Laparoscopy | 4 (11.4%) | 0 (0%) |  |
| Open | 31 (88.6%) | 61 (100%) |  |
| Operation findings, no. (%) |  |  |  |
| Ascites | 7 (20.0%) | 33 (54.1%) | 0.001 |
| Perforation | 0 (0%) | 4 (6.6%) | 0.293 |
| Stoma formation | 1 (2.9%) | 0 (0%) | 0.365 |
| Transfusion, no. (%) | 1 (2.9%) | 8 (13.1%) | 0.149 |
| Blood loss, mean(± SD), ml | 70.00±39.92 | 75.57±49.62 | 0.572 |
| Operation time, mean(± SD), min | 118.14±29.95 | 149.19±39.41 | 0.001 |
| Positive margin, no. (%) | 0 (0%) | 0 (0%) | / |
| No. of retrieved LNs, mean(± SD) | 21.09±9.89 | 20.08±9.68 | 0.629 |
| No. of metastatic LNs, mean(± SD) | 1.86±3.91 | 1.77±2.85 | 0.901 |
| ICU stay, no. (%) | 4 (11.4%) | 21 (34.4%) | 0.013 |
| ICU stay time, mean(± SD), day | 4.25±2.87 | 3.66±2.74 | 0.702 |
| Postoperative complication, no. (%) | 4 (11.4%) | 18 (29.5%) | 0.042 |
| Wound infection | 1 (2.9%) | 5 (8.2%) | 0.411 |
| Pneumonic infection | 2 (5.7%) | 10 (16.4%) | 0.200 |
| Anastomotic leakage | 0 (0%) | 2 (3.3%) | 0.532 |
| Gastric retention | 0 (0%) | 1 (1.6%) | 1.000 |
| MODS | 0 (0%) | 0 (0%) | / |
| AHF | 1 (2.9%) | 0 (0%) | 0.365 |
| 30-days mortality, no. (%) | 0 (0%) | 0 (0%) | / |
| Hospital stay, mean(± SD), day | 8.23±6.50 | 11.13±6.25 | 0.034 |

Abbreviations: *SEMS*, self-expandable metal stents; *LN*, lymph node; *ICU*, intensive care unit; *MODS*, multiple organ dysfunction syndrome; *AHF,* acute heart failure.
